# Circulating Tumor DNA Characteristics Based on Next Generation Sequencing and Its Correlation With Clinical Parameters in Patients With Lymphoma

**DOI:** 10.3389/fonc.2022.901547

**Published:** 2022-07-05

**Authors:** Xiao-Bo Wu, Shu-Ling Hou, Qiao-Hua Zhang, Ning Jia, Min Hou, Wen Shui

**Affiliations:** ^1^ Department of Lymphoma, Cancer Center, Shanxi Bethune Hospital, Shanxi Academy of Medical Sciences, Tongji Shanxi Hospital, Third Hospital of Shanxi Medical University, Taiyuan, China; ^2^ Tongji Hospital, Tongji Medical College, Huazhong University of Science and Technology, Wuhan, China; ^3^ Department of Radiotherapy Abdominal Pelvic Ward Two, Shanxi Provincial Cancer Hospital, Taiyuan, China; ^4^ Department of Emergency, Shanxi Bethune Hospital, Shanxi Academy of Medical Sciences, Tongji Shanxi Hospital, Third Hospital of Shanxi Medical University, Taiyuan, China; ^5^ Department of Cardiopulmonary Function, Shanxi Provincial Cancer Hospital, Taiyuan, China

**Keywords:** lymphoma, tumor heterogeneity, circulating tumor DNA (ctDNA), next-generation sequencing (NGS), gene mutation, prognosis

## Abstract

**Background:**

Lymphoma is a heterogeneous group of tumors in terms of morphological subtypes, molecular alterations, and management. However, data on circulating tumor DNA (ctDNA) mutated genes are limited. The purpose of this study was to investigate the features of the ctDNA mutated genes, the prognosis, and the association between the ctDNA mutated genes and the clinical parameters in lymphoma.

**Methods:**

Differences in the ctDNA between the mutated genes and the prognosis of 59 patients with Hodgkin’s lymphoma (HL) (10.2%), germinal center B-cell–like lymphoma (GCB) (28.8%), nongerminal center B-cell–like lymphoma (non-GCB) (50.8%), and marginal zone lymphoma (MZL) (10.2%) were analyzed by next generation sequencing (NGS) targeting 121 lymphoma-relevant genes.

**Results:**

Genetic alterations were identified in the ctDNA samples with a median of 6 variants per sample. The genetic variation of the ctDNA in the plasma was found to be significantly correlated with the clinical indices in lymphoma. The genetic heterogeneity of different lymphoma subtypes was clearly observed in the ctDNAs from HL, GCB, non-GCB, and MZL, confirming that distinct molecular mechanisms are involved in the pathogenesis of different lymphomas.

**Conclusion:**

Our findings suggest that NGS-based ctDNA mutation analysis reveals genetic heterogeneity across lymphoma subtypes, with potential implications for discovering therapeutic targets, exploring genomic evolution, and developing risk-adaptive therapies.

## Introduction

Lymphoma is a malignant tumor that originates from the lymphopoietic system and is the most common hematologic malignancy in the world. It is divided into Hodgkin’s lymphoma (HL) and non-Hodgkin’s lymphoma (NHL). Lymphoma is a heterogeneous group of tumors in terms of morphological subtypes, molecular alterations, and management, involving a complex diagnosis and management, and different prognoses. There are significant differences in the response of these tumors to standard treatment strategies. Therefore, access to tumor components and genetic material is essential for diagnosis, management, and the selection of targeted therapies.

The prognosis of classical HL has improved with the advancement of novel therapeutic strategies, resulting in a high cure rate (1), and current genomic technologies have also greatly improved the disease classification and prognostication of major subtypes of B-cell lymphomas (2). However, critical clinical needs remain unmet. The estimated 5-year overall survival (OS) was 96.0%–99.4% in the early stages of HL, using the European Organization for Research and Treatment of Cancer staging criteria (3), but the 5-year OS ranges from 42% to 81% only in the advanced-stage disease (4). The combination of rituximab, cyclophosphamide, doxorubicin, vincristine, and prednisolone (R-CHOP) cures approximately 65% of patients with diffuse large B-cell lymphoma (DLBCL). Patients who do not respond to R-CHOP therapy or who experience relapse are treated with a second-line therapy. Long-term remission occurs in 20%–30% of patients but at the cost of high toxicity and treatment-related mortality (5). Therefore, understanding the mechanisms involved and identifying predictive biomarkers is essential.

Tissue biopsy is a traditional method for detecting the molecular features of tumors. However, its limitations are its invasive nature and the difficulty of obtaining serial samples in clinical practice. Given the profound intra-tumor heterogeneity (6, 7), a single-site biopsy is highly unlikely to capture the entire genomic complexity of a tumor. In fact, different regions of the same tumor may show different genetic maps, while biopsies from different parts of the tumor may miss mutations in subclones inhabiting distant sites. Liquid biopsies are based on the analysis of circulating tumor cells (CTCs), circulating tumor DNA (ctDNA), or tumor-derived extracellular vesicles that have been shed from tumors and their metastatic sites into the blood (8). Since ctDNA is derived from tumor cells, it contains tumor-derived genetic alterations that can reflect the molecular heterogeneity of multiple disease sites (9). In the management of lymphoma, genotyping of ctDNA has been successfully integrated into clinical work (10, 11). Next-generation sequencing (NGS) technology has become a promising method for ctDNA mutation profiling due to its high throughput, better sensitivity, and specificity (12).

We analyzed the mutation profiles of different lymphoma subtypes [including HL and B cell non-Hodgkin’s lymphoma (B-NHL)] using patients’ ctDNA and tumor genomic DNA (gDNA). We targeted 121 related genes by NGS to explore the clinical features of ctDNA mutation profiling in lymphomas and reveal the genetic heterogeneity of different subtypes of lymphoma, with the aim of facilitating prognosis predictions and treatment decisions.

## Materials and Methods

### Study Design

From 60 patients with lymphoma who enrolled in the program, 59 patients were included in this retrospective study according to their pathology type. The clinical and follow-up data were collected and the association between them was analyzed. The pathology types included HL (n = 6), germinal center B-cell–like lymphoma (GCB) (n = 17), nongerminal center B-cell–like lymphoma (non-GCB) (n = 30), and marginal zone lymphoma (MZL) (n = 6). The patients were diagnosed with lymphoma between 2019 and 2021 at the Shanxi Bethune Hospital (Taiyuan, China). Of the 59 patients, 21 were aged 65 years or older, the median age was 60 years old, and 26 were male. The exclusion criteria were: (1) patients who have already started any treatment (including steroids) before signing informed consent; (2) patients with contraindications to positron emission tomography; (3) patients who were HIV-positive; (4) patients with hepatitis B or C; (5) pregnant women. All treatments were performed in accordance with the Declaration of Helsinki. The ethics committee of Shanxi Bethune Hospital approved this study. All patients gave informed consent for specimen collection, clinical data collection, and biomarker analysis.

Of the patients, 49.1% had a good performance status (Eastern Cooperative Oncology Group [ECOG] score 0 or 1), most patients (66.1%) presented extranodal involvement, and a minority of patients (30.5%) presented B symptoms. Most of the patients (55.9%) were in Ann Arbor Stage IV. Patients’ demographic and clinical characteristics are summarized in **Table 1**.

**Table 1:** Baseline characteristics of all patients.

Variables	N (%)
Age (years)	
Median	60
Range	24-86
Gender	
Male	26 (44.1)
Female	33 (55.9)
Pathological diagnosis	
HL	6 (10.2)
DLBCL (GCB)	17 (28.8)
DLBCL (non-GCB)	30 (50.8)
MZL	6 (10.2)
Ann Arbor Stage	
I	11 (18.6)
II	7 (11.9)
III	9 (15.3)
IV	32 (54.2)
IPI/IPS	
<2	10 (16.9)
2-4	29 (49.2)
>4	20 (33.9)
ECOG	
0	6 (10.2)
1	23 (38.9)
2	18 (30.5)
3	9 (15.3)
4	3 (5.1)
B symptoms	
Present	18 (30.5)
Absent	41 (69.5)
Extranodal involvement	
With	39 (66.1)
Without	20 (33.9)
Complications	
With	38 (64.4)
Without	21 (35.6)
Ki-67	
<10%	3 (5.1)
10%-50%	7 (11.9)
>50%	49 (83.1)

### Sample Collection and Circulating Tumor DNA Extraction Processing

Plasma samples were collected at baseline. For each patient, 5–10 ml peripheral blood samples were collected within 24 h in ethylenediaminetetra–acetic acid-coated tubes (BD Biosciences). These were centrifuged for 10 min at 3500 rpm at 4°C within 2 h of collection and stored at −80°C. Cell-free DNA (cfDNA) was extracted from 2 ml plasma using the AVENIO cfDNA Isolation Kit (Roche Diagnostics, Mannheim, Germany) and quantified with the Qubit™ dsDNA High Sensitivity Kit (ThermoFisher). Enrichment of the characteristic mononucleosomal fragment peak (160–200 bp) and the absence of contaminating high molecular weight genomic DNA (13, 14) were verified using the Bioanalyzer 2100 High Sensitivity DNA Kit (Agilent Technologies, Santa Clara, CA, USA).

The gDNA was isolated from formalin-fixed paraffin-embedded (FFPE) diagnostic tissue biopsies. Excess paraffin was removed from the FFPE tissue with a scalpel, and the specimens were cut to 10 μm thickness; the first 2–3 exposed and air-exposed slices were discarded, and 1–2 internal slices were reserved for DNA extraction. Sections were immediately placed in 2-ml Eppendorf centrifuge tubes, and DNA was extracted using the FlexiGene DNA kit (Qiagen, Germany) and saved at −80°C for further testing. The DNA content was determined using a NanoDrop™ 2000 ultramicroscopic spectrophotometer (ThermoFisher Scientific, USA).

### Library Construction

The fragment DNA was generated with Bioruptor^®^ (Diagenode,Bioruptor UCD-200) following the manufacturer’s instructions. Libraries were constructed using the KAPA HyperPrep DNA Library Kit (KAPA Biosystem, KK8504). Dual-indexed sequencing libraries were amplified by polymerase chain reaction (PCR) with KAPA HiFi HotStart ReadyMix (KAPA,KK2602) for 4–6 cycles, then cleaned up by purification beads (Corning, AxyPrep FragmentSelect-I Kit, 14223162). Library concentration and quality were determined by the Qubit™ 3.0 system (Invitrogen) and the Bioanalyzer 2100 (Agilent, Agilent HS DNA Reagent, 5067–4627).

### Hybrid Selection and Ultra-deep Next Generation Sequencing

The 5′-biotinylated probe solution was used as the capture probes. The probes for targeted sequencing cover exons and selected introns of 121 lymphoma-related genes. The amplified samples were purified by AMPure XP beads, quantified by quantitative PCR (KAPA) and sized on a Bioanalyzer 2100 (Agilent, Agilent HS DNA Reagent, 5067–4627). Libraries were normalized to 2.5 nM and pooled. Finally, the library was sequenced as paired 150 bp reads on an Illumina HiSeq 4000 according to the manufacturer’s instructions.

### Single Nucleotide Variants and Short Insertions/Deletions Detections

Single nucleotide variants (SNVs) and short insertions/deletions (indels) were identified by VarScan 2 v2.3.9 to generate variant call format files with the minimum variant allele frequency (VAF) threshold set at 0.01 and the p-value threshold for calling variants set at 0.05, with minimum base quality = 20, minimum mapping quality = 1, the minimum coverage = 20, minimum read depth = 8, basic strand-bias filter = 1. All SNVs/indels were annotated with ANNOVAR (version 28) using the filter-based annotation based on human genome hg19 with the database dbscsnv11, and each SNV/indel was manually checked on the integrative genomics viewer (15).

### Statistics

The Chi-squared or Fisher’s exact test was used to compare the samples with certain genetic alterations. A non-parametric test (Mann–Whitney) was used to determine the relationships between different molecular parameters. The correlation between mutated genes and clinical indicators was evaluated by Spearman correlation coefficient. The Kaplan–Meier method and log-rank test were used to analyze the progression-free survival (PFS) rate. The relationship between ctDNA mutations and clinical indicators was analyzed by logistic regression. The Cox proportional hazard regression model was used for univariable analyses. The SPSS™ Statistics version 25.0 software was used for all the statistical analyses, and all graphs were constructed on the Prism version 8.00 (GraphPad Software Inc, USA) and Photoshop CS5 software (Adobe Systems Software Ireland Ltd, Dublin, Ireland). A value of *P* < 0.05 was considered statistically significant.

## Results

### Targeted Next Generation Sequencing Mutation Profiling of Circulating Tumor DNA and Genomic DNA From Patients With Lymphoma

Patients with DLBCL and MZL were treated with the chemotherapy regimen for R-CHOP, and patients with HL were treated with the chemotherapy regimen for Adriamycin, bleomycin sulfate, vinblastine sulfate, and dacarbazine. The time between tissue and liquid biopsy was less than two weeks in all patients (median = 7 days, range 1–12 days).

The ctDNA and tissue biopsies were collected from all patients, and NGS analysis was performed. Patients were considered to have mutations if they had a mutation in their extracted gDNA and/or plasma ctDNA biopsies. The PFS was defined as the time from diagnosis until the date of progression, relapse, death, or the last follow-up. In the present study, variants were found in all patients. A total of 82 genes or sites were identified by genotyping of ctDNA or gDNA collected at diagnosis. In the ctDNA, 52 gene mutations were identified, of which 8 were not found in the corresponding biopsies; whereas gDNA genotyping in tissue biopsies identified 74 gene mutations, of which 30 variants were not found in the corresponding plasma samples. The maximum follow-up time was 33 months.

**Figure 1:**
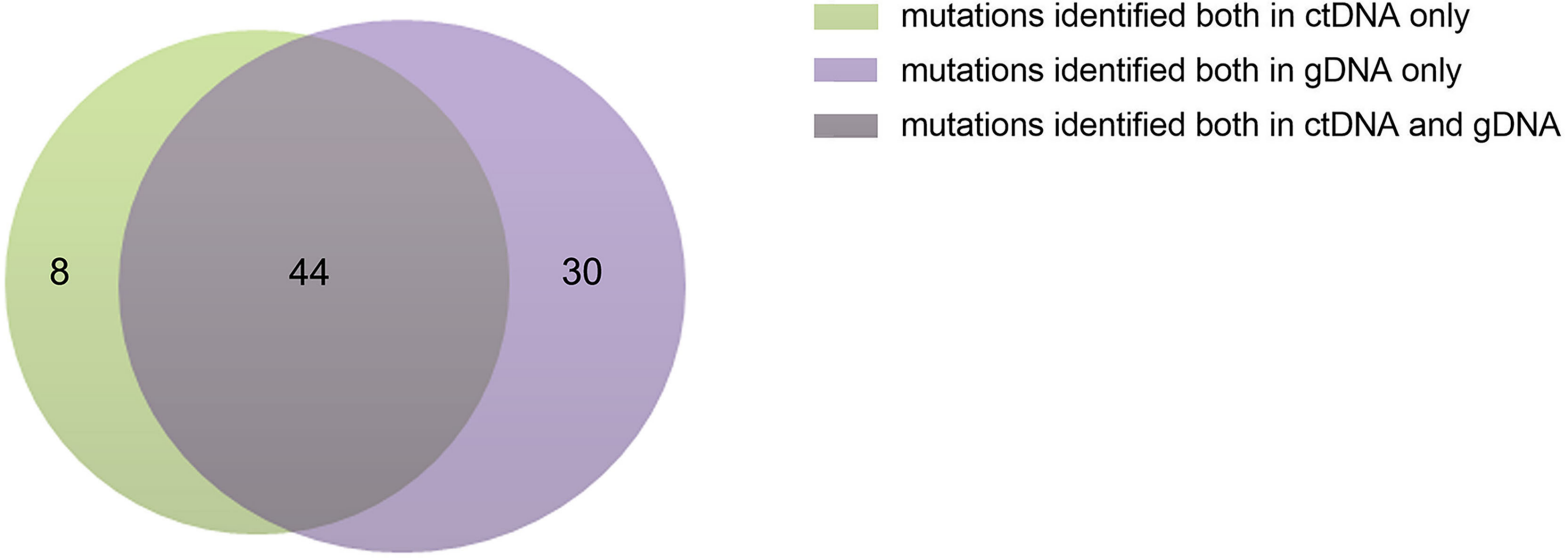
Numbers of ctDNA and gDNA mutations. There were 52 mutations in ctDNA and 74 mutations in gDNA, 44 mutations were common to them.

The concordance of ctDNA samples with biopsy-confirmed tumor mutations was detected in all patients with a kappa value of 0.705 (**Figure 1**), which demonstrated that plasma ctDNA could accurately mirror the profiles of the clones found in tumor tissues.

### Classification and Genotyping of the Patients With Lymphoma

**Figure 2:**
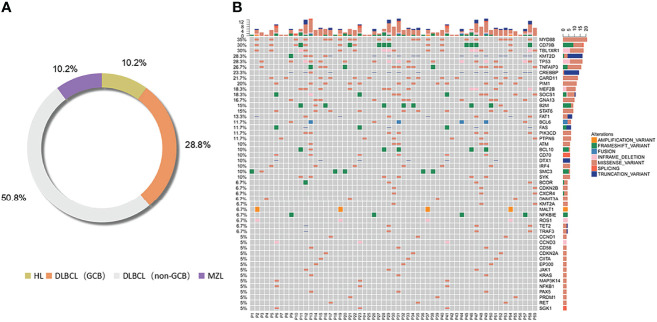
Distribution of lymphoma subtypes. Distribution of pathological subtypes and genetic alterations of ctDNA in the total cohort. **(A)** Detailed distribution of pathological subtypes of 59 lymphomas. **(B)** Genetic alterations of ctDNA in the total cohort. HL, Hodgkin’s lymphoma; DLBCL, diffuse large B cell lymphoma; MZL, marginal zone lymphoma.

According to the classification of cell origin, 6 patients were HL cases (10.2%), 17 patients were GCB cases (28.8%), 30 patients were non-GCB cases (50.8%), and 6 patients were MZL cases (10.2%). The most common subtype was DLBCL (GCB and non-GCB) (47/59, 79.7%). Distribution of lymphoma subtypes is shown in **Figure 2A**.

Genetic alterations were identified in all the ctDNA samples, and the median number of variants was 6 (range 1–16). We divided the most-affected genes of HL, DLBCL, and MZL into 14 specific pathways according to the GeneCards database. Mutations in 14 genes were identified in at least 7 patients. The six most frequently mutated genes identified in the entire group of patients (15/59–21/59, 25.4%–35.6%) were *TNFAIP3*, *MYD88*, *CD79B*, *TBL1XR1*, *TP53*, and *KMT2D*. The ctDNA mutations of different pathological subtypes in patients are shown in **Table 2**. The number of genetic mutations is shown in **Figure 2B**.

**Table 2:** ctDNA mutation in patients of different pathological subtypes.

Subtype	Mutation (median)	Mutation range
HL	2	1-3
DLBCL (GCB)	6	1-10
DLBCL (non-GCB)	6	2-16
MZL	4.5	1-8

**Figure 3:**
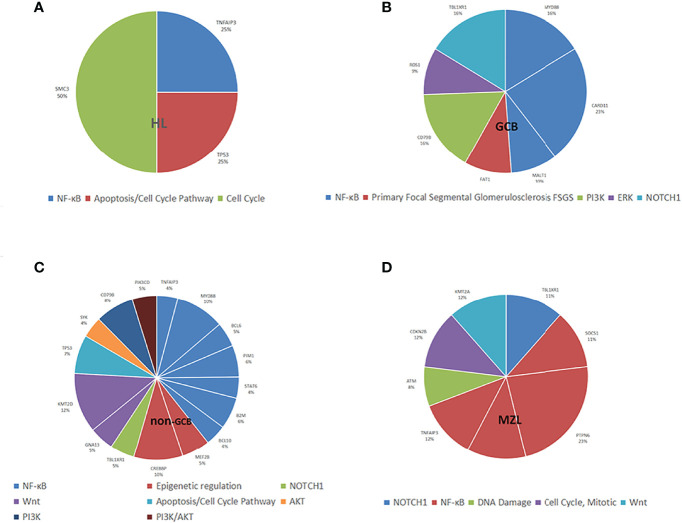
Distribution of mutation allele frequencies and the pathways in lymphoma patients. **(A)** Mutation allele frequencies and the pathways in HL; **(B)** Mutation allele frequencies and the pathways in GCB; **(C)** Mutation allele frequencies and the pathways in non-GCB; **(D)** Mutation allele frequencies and the pathways in MZL.

The mutated genes detected in the ctDNA of patients with HL were *SMC3* (100%), *TNFAIP3* (50.0%), and *TP53* (50.0%). Of these, *SMC3* was a mutation specific to patients with HL. The mutation of genes detected with 20% or higher ratios in patients with GCB included *CARD11* (58.8%), *MYD88* (41.2%), *TBL1XR1* (41.2%), *CD79B* (41.2%), *FAT1* (23.5%), *MALT1* (23.5%), and *ROS1* (23.5%). Mutations of *KMT2D* (56.7%), *MYD88* (46.6%), *CREBBP* (46.7%), *TP53* (36.7%), *CD79B* (36.7%), *PIM1* (30.0%), *B2M* (30.0%), *MEF2B* (26.7%), *TBL1XR1* (23.3%), *STAT6* (20.0%), *BCL6* (23.3%), *GNA13* (23.3%), *PIK3CD* (23.3%), *TNFAIP3* (20.0%), *BCL10* (20.0%), and *SYK* (20.0%) were found in 20% or more of the patients who were diagnosed with non-GCB. Mutations of *MALT1* and *ROS1* were found only in patients with GCB, and the mutations of *TET2* and *TRAF3* were present in patients with non-GCB only. Both GCB and non-GCB showed a significant difference in the mutant allele frequencies of *MALT1*, *CD79B*, *ROS1*, *TBL1XR1*, *PIM1*, *TET2*, and *TRAF3*. Mutations of *PTPN6* (100%), *TNFAIP3*, *TBL1XR1*, *SOCS1*, *CXCR4*, *CDKN2B*, *KMT2A* (all were 50.0%), and *ATM* (33.3%) were found in patients with MZL. Mutation of *TNFAIP3* was common in patients with all subtypes. The gene mutation rate and pathways of each subtype are shown in **Figure 3**. The pathway common to all patients was *NF-κB*.

All four types of lymphoma were associated with tumor inflammation promotion, but HL was mostly characterized by mutations in necroptosis, metabolism, and cell cycle occurrence, and NHL was mostly characterized by mutations in escape immune destruction, cell proliferation, and migration.

### Correlation Between Mutated Genes and Clinical Indicators

**Figure 4:**
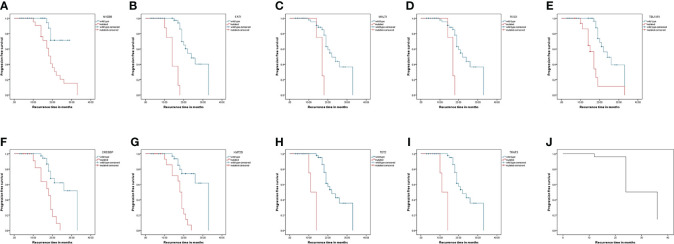
Progression-free survival curves of patients with lymphoma. Kaplan-Meier curves for progression-free survival by presence or absence of genes mutation. **(A–I)** was genes: MYD88, FAT1, MALT1, ROS1, TBL1XR1, CREBBP, KMT2D, TET2, and TRAF3. **(J)** was the progression-free survival curve for all patients.

The Kaplan–Meier analysis showed that mutations in *MYD88*, *FAT1*, *MALT1*, *ROS1*, *TBL1XR1*, *CREBBP*, *KMT2D*, *TET2*, and *TRAF3* were significantly different for the progression of the patients. Their survival curves are shown in **Figure 4**.

To analyze the correlations between the *MYD88*, *FAT1*, *MALT1*, *ROS1*, *CREBBP*, *KMT2D*, *TET2*, and *TRAF3* mutations and the clinical parameters of patients with lymphoma, we divided the mutations into positive and negative. **Table 3** summarizes the correlations of *MYD88*, *FAT1*, *MALT1*, *ROS1*, *CREBBP*, *KMT2D*, *TET2* and *TRAF3* mutations with the clinical parameters of patients with lymphoma, including gender, age, B symptoms, extranodal involvement, ECOG, and complications. The Chi-squared test showed that the mutation of *MYD88* had a significant correlation with ECOG score 3–4 and complications, and the mutations of *MALT1* or *ROS1* had a significant correlation with ECOG score 3–4. The mutations of *CREBBP* or *KMT2D* had a significant correlation with age >65.5 years. The mutation of *TET2* or *TRAF3* had a significant correlation with complications. Mutations in *TBL1XR1* are not listed since they did not correlate with clinical parameters.

**Table 3:** Correlation of MYD88, FAT1, MALT1, ROS1, CREBBP, KMT2D, MALT1 and ROS1 mutations with clinical parameters of lymphoma patients.

Clinical Parameters	n	MYD88 mutations		χ^2^	*P*	FAT1 mutations		χ^2^	*P*	MALT1 or ROS1 mutations		χ^2^	*P*
		+	-			+	-			+	-		
Sex				0.167	0.683			0.162	0.687			0.075	0.784
Male	26	10	16			3	23			1	25		
Female	33	11	22			5	28			3	30		
Age (years)				0.058	0.810			2.569	0.109			0.656	0.418
>65.5	18	6	12			0	18			0	18		
≤65.5	41	15	26			8	33			4	37		
B symptoms				2.020	0.155			0.604	0.437			0.000	1.000
Present	18	4	14			1	17			1	17		
Absent	41	17	24			7	34			3	38		
Extranodal involvement				3.209	0.073			3.157	0.076			0.877	0.349
With	39	17	22			8	31			4	35		
Without	20	4	16			0	20			0	20		
ECOG				4.757	0.029			3.131	0.077			11.946	0.001
0-2	47	13	34			4	43			0	47		
3-4	12	8	4			4	8			4	8		
Complications				3.894	0.048			0.269	0.604			0.998	0.318
With	38	17	21			4	34			4	34		
Without	21	4	17			4	17			0	21		
Clinical Parameters	n	CREBBP mutations		χ^2^	*P*	KMT2D mutations		χ^2^	*P*	TET2 or TRAF3 mutations		χ^2^	*P*
		+	-			+	-			+	-		
Sex				0.011	0.917			0.746	0.388			0.000	1.000
Male	26	6	20			6	20			2	24		
Female	33	8	25			11	22			2	31		
Age (years)				12.077	0.001			9.031	0.003			0.656	0.418
>65.5	18	10	8			10	8			0	18		
≤65.5	41	4	37			7	34			4	37		
B symptoms				0.263	0.608			1.863	0.172			0.656	0.418
Present	18	3	15			3	15			0	18		
Absent	41	11	30			14	27			4	37		
Extranodal involvement				0.649	0.421			0.021	0.885			0.877	0.349
With	39	11	28			11	28			4	35		
Without	20	3	17			6	14			0	20		
ECOG				0.246	0.620			0.001	0.976			0.163	0.687
0-2	47	10	37			13	34			4	43		
3-4	12	4	8			4	8			0	12		
Complications				0.095	0.758			1.516	0.218			5.043	0.025
With	38	10	28			13	25			0	38		
Without	21	4	17			4	17			4	17		

## Discussion

With the development in NGS technology, a comprehensive exploration of the somatic alterations within ctDNA has become increasingly accessible. The great sequencing depth used for ultra-deep sequencing makes it very powerful for profiling clinical samples, such as formalin fixed paraffin embedded and ctDNA. Greater depth of coverage also allows to pick out mutations present only in a small fraction of malignant cells. However, accurate variant calling remains challenging due to variable coverage, sequencing errors, alignment artifacts, and other issues. Lower tumor purity proportionally reduces the effective coverage of the variant alleles in tumor cells, reducing detection sensitivity (16). Bioinformatics tools mad it possible to detect VAFs of 1% or even lower. VarScan 2 performed best overall with sequencing depths of 100× and 1000× required to accurately identify variants present at 10% and 1%, respectively (17). The minimum VAF for detection of a sequence variant is not highly correlated with the percentage tumor cellularity of the specimen or the percentage of tumor cells that harbor the sequence change. In the setting of detecting minimal residual disease, accurate detection of VAFs substantially <0.01 may be required (18), with VAF sufficiently detected as low as 0.1–0.2% (19). In the study, the minimum VAF threshold was set at 0.01, thus VarScan 2 identified the variants accurately.

Our understanding of lymphoma is rapidly evolving, driven by advances in single-cell technology. Although studies have revealed some similarities between different subtypes of lymphoma, they still face challenges in terms of tumor heterogeneity. Our study performed a targeted panel sequencing of 59 patients with lymphoma on 121 key genes and analyzed their genetic alterations. Furthermore, previous studies had proved the pre-analytical stability of ctDNA under different storage conditions (20, 21), and NGS-based ctDNA analysis could reflect genetic heterogeneity among different lymphoma subtypes, indicating that ctDNA could be a noninvasive and feasible biomarker for patients with lymphoma. Analysis of ctDNA in the plasma is clinically used to identify actionable mutations, detect residual or recurrent disease and can assess the mutational heterogeneity of the entire tumor cell population. However, ctDNA analysis cannot address mutations within individual cells and cannot assess cancer phenotypes, such as the expression of drug targets and protein biomarkers. Given the heterogeneity, the fact that resistant clones of tumors may represent only a small proportion of the entire tumor and are unlikely to suffer apoptosis, the genomes of resistant tumor subclones may not be detectable at the current sensitivity limits of cell-free DNA assays. As intact cancer cells that have entered the blood, CTCs show the predictive capability of the response to drugs through analyzing protein biomarkers on CTCs and show the broad detection of mutations through genome-wide sequencing (22). CTCs are identified and sequenced to identify operable mutations in drug-resistant subclones that are not present in the majority of tumors to guide subsequent therapy. In addition, single-cell sequencing of CTC provides better access to variability between clones with different drug resistance mechanisms. Therefore, CTCs are better suited to study heterogeneity at the cellular level.

It has been reported that *MYD88* mutation was detected in the cfDNA of one patient with lymphoplasmacytic lymphoma (23). Schmitz et al. (24) studied 574 DLBCL biopsy samples using exome and transcriptome sequencing and identified four prominent genetic subtypes in DLBCL, one of which was termed “MCD” (based on the co-occurrence of *MYD88^L265P^* and *CD79B* mutations). Analysis of genetic pathways suggested that MCD relied on “chronic active” B-cell receptor signaling that is amenable to therapeutic inhibition. Our study showed similar findings that *MYD88* was the most frequently mutated gene identified in the ctDNA of patients, followed by *CD79B*, and both mutations occurred in patients with DLBCL, but not in patients with HL or MZL. This indicates that the mechanism of DLBCL development is vastly different compared with HL and MZL, and *MYD88* and *CD79B* mutations might be a major driver of DLBCL development.

Venturutti et al. (25) found through studies in mice that *TBL1XR1* alterations lead to a striking extranodal immunoblastic lymphoma phenotype that mimics the human disease. Jangam et al. (26) performed targeted deep sequencing of 8 ocular adnexal mucosa-associated lymphoid tissue lymphoma (OAML) cases, and identified *TBL1XR1* as recurrently mutated in OAML (4/8), where cases of OAML with mutations in *TBL1XR1* showed equivalent or increased vascular density compared with cases without mutations in *TBL1XR1*. Wang et al. (27) found that patients with primary testicular lymphoma with the *TBL1XR1* mutation had an inferior OS than patients with *TBL1XR1* wild type, irrespective of treatment therapy. Consistent with those studies, the present study found that patients with mutations in the *TBL1XR1* gene had significantly lower PFS rates than those without mutations, both in the population of patients with NHL and in the overall population of patients with lymphoma.

It is well known that mucosa-associated lymphoid tissue lymphoma translocator protein 1 (*MALT1*), a key adaptor protein regulating the *NF-κB* pathway, is the only protease in the pathogenesis of these related diseases. In the present study, *MALT1* mutations in the ctDNA were also found in patients with lymphoma and were only found in patients with GCB. Univariate analysis revealed that patients with *MALT1* gene mutation had a significantly lower PFS rate than those with the wild-type *MALT1* gene (28). Therefore, *MALT1* could be a target for the treatment of GCB (29).

Nie et al. found that *CREBBP* and *EP300* genes are two frequently mutated epigenetic regulators in B-cell lymphoma and that synthesis between them is lethal (30). Mosquera et al. (31) found that mutations in *CREBBP*, *TNFRSF14*, and *KMT2D* were mainly found in follicular lymphoma, while mutations in *BTG2*, *HTA-A*, and *PIM1* were more frequent in DLBCL. In the present study, *CREBBP* and *KMT2D* appeared in patients with non-GCB, and inconsistently, *CREBBP* and *KMT2D* were mutated more frequently in patients with non-GCB than in *PIM1*. This illustrates the heterogeneity of lymphoma; there was still a high degree of heterogeneity in lymphomas of the same pathological type.

The *ROS1* fusion proteins resulting from chromosomal rearrangements of the *ROS1* gene are targetable oncogenic drivers in diverse cancers (32). Inflammatory myofibroblastic tumor fusions involving *ROS1*, *PDGFRβ*, *RET*, and *NTRK* have also been described in inflammatory myofibrosarcoma (33). Over the past few years, inhibitors of the c-Ros oncogene 1 (*ROS1*) have been approved and are currently used in clinical practice in patients with advanced non-small cell lung cancer (34, 35). However, *ROS1* mutations have not been reported in lymphoma. In the present study, as with *MALT1*, *ROS1* mutation was only found in GCB. This means that GCB has a unique *ROS1* mutation, which had a different mechanism of occurrence from other DLBCL.

Esther et al. (36)found that miR-92a and TET2 may play a synergistic role in the pathogenesis of NHL malignancies. Oreofe et al. (37)found that TET2 mutations occurred in 76% of patients with angioimmunoblastoma T-cell lymphoma (AITL). The bridging protein TNF receptor-associated factor 3 (TRAF3), as a tumor suppressor, is a key regulator of B-lymphocyte survival, and TRAF3 deficiency is sufficient to metabolically reprogram B cells (38). In this study, TET2 and TRAF3 were found to be present only in non-GCB patients, suggesting that non-GCB has a unique pathogenesis that distinguishes it from GCB and HL.

In the study, we found that all four types of lymphoma are associated with the promotion of tumour inflammation. It is well known that cancer cells, as well as surrounding stromal and inflammatory cells, are involved in carefully orchestrated interactions to form an inflammatory tumour microenvironment (TME). Cells within the TME are highly plastic, constantly changing their phenotypic and functional characteristics (39). However, each subtype has its own characteristics.

Dysregulation of apoptotic cell death mechanisms is a hallmark of cancer. Altered apoptosis is not only responsible for tumor development and progression, but also for tumor resistance to therapy. In contrast, defects in the death pathway may lead to drug resistance, thereby limiting the effectiveness of treatment (40). Therefore, a better understanding of mutations in the apoptotic pathway could improve the efficacy of cancer therapy and bypass resistance. In this study, apoptosis pathway was found in HL and non-GCB patients, which means that the population may not respond well to certain treatments and new therapeutic strategies need to be developed to counter their resistance to drugs.

In addition, the pathways in which the mutated genes are located reveal that some mutated genes in non-GCB patients are associated with epigenetic inheritance, which is completely different from the other three types. In contrast to genetic changes, epigenetic changes are reversible (41). This constitutes a promising area to understand the role of epigenetic alterations in cancer and to find new alternatives to traditional strategies (42).

Studies have shown that ECOG is an independent prognostic factor for secondary malignancies after surgery for gastrointestinal or gynecological tumors (43). ECOG is also an independent factor in the OS of patients with early onset colorectal cancer (44). In our study, we found ECOG score 3–4 was closely associated with mutations in *MYD88*, *MALT1*, and *ROS1*, which suggested that ECOG might be associated with lymphoma heterogeneity. In addition, the presence of complications was also associated with *MYD88*, *TET2*, and *TRAF3* mutations, which suggested that mutations in these genes might influence the occurrence of other complications. Furthermore, we found that mutations in the *CREBBP* and *KMT2D* genes were strongly correlated with the age of the patients, and the rate of mutations in these genes was significantly higher in patients over 65.5 years than in those under 65.5 years. Therefore, patients with co-morbidities, higher ECOG and age over 65.5 years are strongly associated with genetic mutations.

However, even though ctDNA has some advantages for patients, the potential for loss of information and the associated risks are still considered a challenge. We found a discrepancy in mutation comparisons between gDNA (FFPE samples) and ctDNA (liquid biopsies), which may lead to false-negative and false-positive results in ctDNA analysis. This is related to technical and biological factors (45). As the total number of genomic copies in the plasma volume of a sample is very limited, the number of specific variants of interest is also very limited. Also, some false negative results simply cannot be prevented, due to biological factors such as low DNA shedding in certain tumours or the location of the metastases themselves (46). In addition, multiple mutation enrichment methods and additional steps for error suppression strategies are required due to the risk of introducing errors in the library preparation or sequencing process itself (47, 48).

The stability of ctDNA varies under different conditions. Qing Kang in 2016 had processed the plasma of ten patients with metastatic breast cancer after 2, 6, and 48 h post-collection, and found that ctDNA stable for up to 6 h in both Streck and ethylenediaminetetraacetic acid (EDTA) tubes, and, one out of four patients with detectable ctDNA showed a ~ 50% decline in ctDNA in the EDTA tube after 48 h (49). Emanuela Henao Diaz found that the ctDNA levels at zero hours were not significantly different to 24- or 48-hour *in vitro* incubation in any investigated condition (50). In 2018, American Society of Clinical Oncology and College of American Pathologists jointly reviewed the information about clinical ctDNA assays and provided a framework for future research, and indicated that testing for ctDNA was optimally performed on plasma collected in cell stabilization or EDTA tubes, with EDTA tubes processed within 6 h of collection (51). Taken together, it is a current consensus that ctDNA is stable within 6 h after the sample collection. To preserve the stability of ctDNA, we used ethylenediaminetetraacetic acid-coated tubes to collect peripheral blood samples from patients and preserved them by centrifugation within 2 hours. At the same time, an ultrasensitive method was used to detect mutations and copy number changes to ensure the stability of ctDNA in the blood stream and to reduce the errors caused by the assay.

There were also limitations to our study. In this study, the ctDNA concentration was not involved, and only the mutation abundance was detected. Since mutation abundance was not related to the ctDNA concentration, and the data were quality controlled, so the accuracy of the data could be guaranteed. In addition, the follow-up period of up to 33 months is not sufficient to demonstrate a correlation between mutations and clinical features, and a longer follow-up period is needed in future studies.

## Conclusion

In summary, we found that ROS1 mutations were uniquely present in GCB, while TET2 and TRAF3 were only present in non-GCB, and both MYD88 and CD79B mutations appeared only in DLBCL patients. All four types of lymphomas were associated with promotion of tumor inflammation, whereas apoptotic pathways were present only in patients with HL and non-GCB. NGS-based ctDNA mutation profiling revealed the biology of lymphoma and could identify mutational differences among lymphoma subtypes, which was a promising approach for exploring genomic evolution and discovering potential therapeutic targets, thereby facilitating personalized treatment. Future studies will require single-cell sequencing of CTCs to reveal the tole of relevant mutations in different subclones and drug resistance mechanisms.

## Data Availability Statement

The original contributions presented in the study are included in the article/supplementary material. Further inquiries can be directed to the corresponding author.

## Ethics Statement

The studies involving human participants were reviewed and approved by Ethics Committee of Shanxi Bethune Hospital(YXLL-KY-2021-011). The patients/participants provided their written informed consent to participate in this study.

## Author Contributions

Conception and design of the research: X-BW Acquisition of data: X-BW, NJ, WS. Analysis and interpretation of the data: X-BW. Statistical analysis: X-BW, MH. Writing of the manuscript: X-BW. Critical revision of the manuscript for intellectual content: X-BW, S-LH, Q-HZ. All authors read and approved the final draft.

## Conflict of Interest

The authors declare that the research was conducted in the absence of any commercial or financial relationships that could be construed as a potential conflict of interest.

## Publisher’s Note

All claims expressed in this article are solely those of the authors and do not necessarily represent those of their affiliated organizations, or those of the publisher, the editors and the reviewers. Any product that may be evaluated in this article, or claim that may be made by its manufacturer, is not guaranteed or endorsed by the publisher.

## Abbreviations

HL, Hodgkin’s lymphoma; NHL, non-Hodgkin’s lymphoma; cHL, classical Hodgkin’s lymphoma; OS, overall survival; DLBCL, diffuse large B-cell lymphoma; CTCs, circulating tumor cells; ctDNA, circulating tumor DNA; NGS, Next-generation sequencing; GCB, germinal center B-cell-like lymphoma; non-GCB, non-germinal center B-cell-like lymphoma; MZL, marginal zone lymphoma; gDNA, Tumor genomic DNA; SNVs, Single nucleotide variants; VCF, Variant Call Format; IGV, Integrative Genomics Viewer; MAFs, mutant allele frequencies; PTL, primary testicular lymphoma; NSCLC, non-small cell lung cancer.
